# Surgical management of tuberculous epididymo-orchitis: a retrospective study of 81 cases with long-term follow-up

**DOI:** 10.1186/s12879-021-06753-w

**Published:** 2021-10-15

**Authors:** Yin Huang, Bo Chen, Dehong Cao, Zeyu Chen, Jin Li, Jianbing Guo, Qiang Dong, Qiang Wei, Liangren Liu

**Affiliations:** 1 Department of Urology, Institute of Urology, West China Hospital, Sichuan University, Guoxue Alley, No. 37, Chengdu, 610041 Sichuan People’s Republic of China; 2 West China School of Medicine, Sichuan University, Chengdu, China

**Keywords:** Tuberculosis, Epididymo-orchitis, Chemotherapy-surgery-pharmacotherapy, Follow up, Sexual function, Fertility

## Abstract

**Background:**

Nowadays, most studies of tuberculous epididymo-orchitis (TBEO) are case reports or small sample cohort series. Our study is aimed to present the largest series of TBEO with our management experiences and long-term follow-up outcomes.

**Methods:**

Patients diagnosed with TBEO after surgical procedures at Department of Urology, West China Hospital from 2008 to 2019 were included. All clinical features, auxiliary examination results, treatment and histopathological findings were extracted if available.

**Results:**

Eighty-one patients (mean age 50.77 ± 16.1 years) were included. Scrotal swelling (N = 47, 58.0%) and pain (N = 29, 35.8%) were the most common presenting complaint. Pyuria and microscopic hematuria were observed in twenty-two (27.2%) and eight patients (9.9%), respectively. Urine acid fast bacilli cultures were available in 16 patients and all were negative. The mean duration between the onset of symptoms and the definite diagnosis was 6.42 ± 7.0 months. TBEO was considered in 30 (37.0%), tumors in 28 (34.6%) and nonspecific bacterial epididymo-orchitis in 23 (28.4%) patients. All patients received triple therapy of chemotherapy-surgery-pharmacotherapy and definite diagnosis was confirmed through histopathology of surgical specimens. Fifty-five patients were followed up regularly (mean follow-up 82.35 ± 36.6 months). One patient (1.2%) died from liver cirrhosis and no recurrence was observed. Postoperative complications included erectile dysfunction in 4 patients (4.9%), premature ejaculation in 5 patients (6.2%) and sterility in 7 patients (8.6%).

**Conclusions:**

We recommend patients with advanced TBEO to receive triple therapy of chemotherapy-surgery-pharmacotherapy. Physicians should pay more attention to patients’ sexual function and fertility during follow up after treatment completed.

## Background

According to the 2019 World Health Organization global tuberculosis (TB) report, about 10 million (range, 9.0–11.1 million) new cases of TB were reported worldwide in 2018. There were an estimated 1.2 million TB deaths among HIV-negative people and an additional 251,000 deaths among HIV positive people in 2018 [1]. TB can affect people of both sexes in all age groups but the highest burden is in men (aged ≥ 15 years), who accounted for 57% of all TB cases, while women and children (aged < 15 years) accounted for 32% and 11%, respectively. China is the second highest TB-burden country after India, accounting for 9% of global TB cases in 2018 [1]. In addition to lymphatic involvement, urogenital TB is the most common manifestation of extrapulmonary TB and is more frequent in middle-aged men, which accounts for 33.7–45.5% of the extrapulmonary TB worldwide [2]. Compared with renal TB, male genital TB is a rare subtype of the urogenital TB, which can be classified as TB epididymitis, TB orchitis, TB of the prostate, TB of the seminal vesicles, and TB of the penis [2, 3].

Since the lack of preoperative diagnostic methods with high sensitivity and specificity, tuberculous epididymo-orchitis (TBEO) with nonspecific clinical signs is often misdiagnosed with bacterial infection or tumor [4]. Standard anti-tuberculosis chemotherapy is the first-line therapy for TBEO. However, surgical intervention may be unavoidable in cases of hard to diagnose, or poorly responding to chemotherapy [5–7]. Patients with TBEO received regular anti-tuberculosis chemotherapy plus surgery intervention are seldom to recur [4, 8]. Nowadays, the majority of studies of TBEO are case reports or small sample cohort series including less than 50 cases. Furthermore, long-term follow-up data after treatment are deficient [4–6, 8]. Therefore, our study is aimed to present our experiences on the clinicopathological characteristics, management and 11-year follow-up outcomes of TBEO at a large medical center in west China.

## Methods

### Setting and study design

From January 2008 to May 2019, patients diagnosed with TBEO after surgical procedures at Department of Urology, West China Hospital were included in our retrospective observational study. All clinical features (symptoms, physical signs, duration of disease, past medical history, comorbidities and organ involvement), auxiliary examination results (hemogram, erythrocyte sedimentation rate (ESR), C-reactive protein (CRP), urinalysis, blood biochemistry, tumor markers, urine culture, ultrasonography and radiology), treatment (drug therapy and surgery) and histopathological findings (microscopy, Ziehl–Neelsen acid fast stain and polymerase chain reaction (PCR)) were retrieved from medical records if available. Patients who only received drug therapy without surgery were excluded.

### Diagnosis

TBEO was definitely diagnosed in the presence of clinical findings combined with one of the following criteria: (1) Positivity of acid fast bacilli (AFB) in urine. (2) Positive urine culture for *M. tuberculosis*. (3) Positivity of PCR for *M. tuberculosis* in urine. (4) Typical granulomatous inflammation with caseous necrosis in microscopy plus any positivity of Ziehl–Neelsen acid fast stain or PCR for *M. tuberculosis* in any relevant tissue specimen [9]. In our institution, histopathological evidence of TBEO was only obtained from postoperative histopathological findings of surgical specimens, no preoperative histopathological examination (e.g. fine-needle aspiration cytology (FNAC)) was performed. According to the criteria of our institution: ESR > 20 mm/1 h, CRP > 5 mg/L, serum beta-human chorionic gonadotropin (β-HCG) ≥ 3.81 mIU/ml, serum alpha-fetoprotein (AFP) ≥ 8 ng/ml and serum lactate dehydrogenase (LDH) ≥ 220 U/L were considered to be elevated.

### Treatment and follow up

Surgical indications for patients with TBEO in our center including: (1) Regular anti-tuberculosis pharmacotherapy for 1–2 months was completed but tuberculous lesions were still not controlled. (2) TBEO was diagnosed in advanced stage with widely spread of *M. tuberculosis*. (3) Tuberculous complications including hydrocele, abscess, sinus or fistula were observed. (4) Clinical diagnosis of scrotal tumors was suspected. (5) Clinical diagnosis was nonspecific bacterial epididymo-orchitis but the efficacy of antibiotic treatment was limited. Patients with clinical diagnosis of TBEO received anti-tuberculosis chemotherapy for 2–4 weeks before surgery. Surgical procedure (orchiectomy, epididymectomy or epididymo-orchidectomy) and postoperative anti-tuberculosis therapy with 4 drugs (rifampicin, isoniazid, ethambutol and pyrazinamide) for 6–12 months were performed in all patients. After completion of the therapy, patients were followed up for symptoms, physical examination, scrotal ultrasound, sexual function and fertility until June, 2020.

### Statistical analysis

The data were analyzed by the IBM SPSS Statistics (version 25). Continuous variables were expressed as mean ± standard deviation (SD) or median with interquartile range (IQR). *P* value < 0.05 was considered as statistically significant.

## Results

### Clinical characteristics

During the 11 years period of the study, a total of 98 cases were recorded as TBEO. After exclusion of those not meeting the diagnostic criteria (N = 11), repeating records (N = 2) and patients who did not undergo surgery (N = 4), 81 separate patients with TBEO were included in our study. Clinical characteristics including symptoms, physical examination, urinalysis, laboratory findings and history of TB of 81 patients are shown in Table 1.

**Table 1:** Clinical characteristics of 81 patients

Characteristics	No. Pts/total (%)
Ages (years)	
< 60	62/81 (76.5)
≥ 60	19/81 (23.5)
Symptoms	
Scrotal swelling	47/81 (58.0)
Scrotal pain	29/81 (35.8)
Fever	6/81 (7.4)
Night sweats	1/81 (1.2)
Weight loss	11/81 (13.6)
Frequency / urgency	2/81 (2.5)
Dysuria	3/81 (3.7)
Physical examination	
Left scrotal mass	16/81 (19.8)
Right scrotal mass	27/81 (33.3)
Bilateral scrotal mass	4/81 (4.9)
Abscess	2/81 (2.5)
Sinus	1/81 (1.2)
Urinalysis	
Pyuria	22/81 (27.2)
Microscopic hematuria	8/81 (9.9)
Positive urine AFB culture	0/16 (0)
Laboratory findings	
Increased ESR	7/14 (50.0)
Increased CRP	5/8 (62.5)
Increased WBC	4/81 (4.9)
Increased serum AFP	3/30 (10.0)
Increased serum β-HCG	1/30 (3.3)
Increased serum LDH	10/81 (12.3)
Anemia	8/81 (9.9)
Hypoalbuminemia	12/81 (14.8)
History of TB	10/81 (12.3)
Lung	8/81 (9.9)
Kidney	1/81 (1.2)
Bone	1/81 (1.2)

*AFB* acid fast bacilli, *ESR* erythrocyte sedimentation rate, *CRP* C-reactive protein, *WBC* white blood cell, *AFP* alpha-fetoprotein, *β-HCG* beta-human chorionic gonadotropin, *LDH* lactate dehydrogenase, *TB* tuberculosis

The average age of the patients was 50.77 ± 16.1 years (range, 13–90 years). Scrotal swelling (N = 47, 58.0%) was the most common presenting complaint, followed by scrotal pain (N = 29, 35.8%). Unilateral and bilateral scrotal mass were detected through physical examination in 43 (53.1%) and 4 (4.9%) patients, respectively. The most positive findings of urinalysis were pyuria (N = 22, 27.2%) and microscopic hematuria (N = 8, 9.9%). AFB culture results were available in only 16 patients and all were negative. The duration between the onset of symptoms and the definite diagnosis varied from 0.3 to 36 months, with an average duration of 6.42 ± 7.0 months. Furthermore, TB history of other organs was reported in ten patients (12.3%). Besides, fifteen patients (18.5%) were diagnosed with TBEO in other medical centers before registered in our hospital and had received regular anti-tuberculosis pharmacotherapy for 1–12 months, but the scrotal tuberculosis lesions of these patients were not controlled effectively.

Abnormal rates of imaging findings in 81 patients are shown in Table 2. Scrotal ultrasound detected the signs of epididymo-orchitis in 30 of 39 patients. Forty-one patients received computed tomography (CT) scan and infectious disease was observed in 36 patients. Magnetic resonance imaging (MRI) was performed in 2 patients and both found an abnormality. Chest radiological evidence of pulmonary infection was found in 43 of 81 patients, and 6 patients (7.4%) had and evidence of active pulmonary TB. In addition, ten cases (12.3%) of hydrocele and four cases (4.9%) of varicocele were confirmed by scrotal imaging.

**Table 2:** Abnormal rates of imaging findings in 81 patients

Imaging modality	No. Pts (total)	Percentage (%)
Ultrasonography	30 (39)	76.9
Computed tomography	36 (41)	87.8
Magnetic resonance	2 (2)	100
Chest radiography	43 (81)	53.1

### Clinical diagnosis and treatment

Before surgery, thirty patients (37.0%) were clinically diagnosed with TBEO, while 28 patients (34.6%) were diagnosed with tumors. Twenty-three patients (28.4%) were preoperatively diagnosed with nonspecific bacterial epididymo-orchitis but routine antibiotic treatment was noneffective (Table 3). Surgical procedure was performed for all patients. Thirty patients with clinical diagnosis of TBEO received preoperative anti-tuberculosis chemotherapy for 2–4 week. Orchiectomy, epididymectomy and epididymo-orchidectomy were performed in two (2.5%), twenty-seven (33.3%) and fifty-two (64.2%) patients, respectively. Seventy-five patients (92.6%) received unilateral orchiectomy or epididymectomy. Bilateral surgical procedure was performed in 6 patients (7.4%) (Table 3). Scrotal masses were found in 43 patients (53.1%) in surgery, with the mean diameter of 3.18 ± 1.6 cm (range, 0.2–7.0 cm).

**Table 3:** Clinical diagnosis, treatment and histopathology characteristics of 81 patients

Characteristics	No. Pts (total)	Percentage (%)
Clinical diagnosis		
TBEO	30 (81)	37.0
Tumor	28 (81)	34.6
Nonspecific bacterial epididymo-orchitis	23 (81)	28.4
Surgical procedure		
Unilateral	75 (81)	92.6
Bilateral^a^	6 (81)	7.4
Orchiectomy	2 (81)	2.5
Epididymectomy	27 (81)	33.3
Epididymo-orchidectomy	52 (81)	64.2
Histopathology		
Positive acid fast stain	62 (77)	80.5
Positive PCR	24 (43)	55.8
Any of the above	81 (81)	100
Granulomatous inflammation with caseous necrosis	81 (81)	100
Isolated testicular TB	23 (81)	28.4
Isolated epididymal TB	31 (81)	38.3
Testicular and epididymal TB	27 (81)	33.3

*TBEO* tuberculous epididymo-orchitis, *PCR* polymerase chain reaction, *TB* tuberculosis

^a^One patient underwent bilateral orchiectomy, one patient underwent bilateral epididymectomy, and four patients underwent orchiectomy and contralateral epididymectomy

### Histopathological findings

**Fig. 1:**
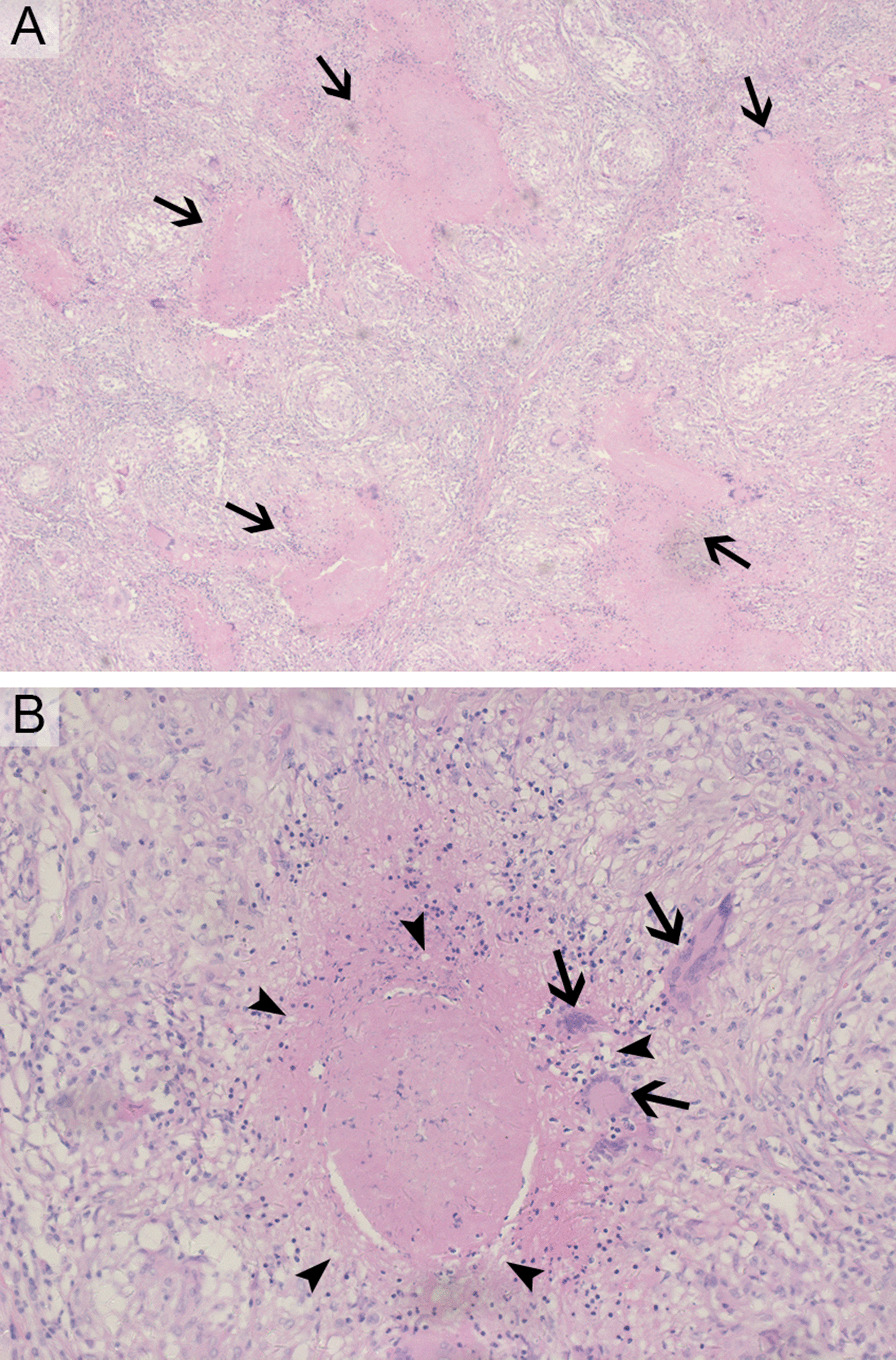
Microscopy Images of Tuberculous Granulomas in Hematoxylin and Eosin Stain. At low magnification (**A**), the typical granulomatous inflammation with amorphous caseous necrosis (arrows) were observed. At high magnification (**B**), the amorphous caseous necrosis was surrounded by granulomas contain epithelioid histiocytes (arrowheads), Langhans giant cells (arrows) and lymphocytes

Postoperative histopathology showed the typical granulomatous inflammation with amorphous caseous necrosis in all surgical specimens (Fig. 1A). At high magnification, the amorphous caseous necrosis was surrounded by granulomas contain epithelioid histiocytes, Langhans giant cells and lymphocytes (Fig. 1B). Ziehl–Neelsen acid fast stain was performed in 77 specimens and was positive in sixty-two (80.5%). PCR findings for *M. tuberculosis* identification were available in 43 surgical specimens and twenty-four cases (55.8%) showed a positive result. Results of histopathological findings are shown in Table 3.

### Prognosis

All patients were followed up regularly until June, 2020 except 26 patients (32.1%) due to loss of contacts. Follow-up ranged from 14 months to 11.5 years, with an average follow-up of 82.35 ± 36.6 months. During follow up, only 1 patient (1.2%) died from liver cirrhosis and no recurrence was observed. Symptoms such as fever, scrotal pain and irritative urinary symptoms were under control in 47 cases (58.0%). Urinalysis parameters and scrotal imaging were stable in 50 patients (61.7%). Postoperative complications included erectile dysfunction in 4 patients (4.9%), premature ejaculation in 5 patients (6.2%) and sterility in 7 patients (8.6%).

## Discussion

Until now, *M. tuberculosis* was the most frequently isolated species in humans all over the world, followed by *M. bovis* [10]*.* However, the species *M. tuberculosis* has been inaccurately used to represent the Mycobacterium tuberculosis complex (MTBC), including *M. tuberculosis*, *M. africanum*, *M. bovis*, *M. canettii* and so on [10, 11]. As one of the most virulent pathogens for humans, *M. tuberculosis* has a slow replication rate, which accounts for the latent nature of the infection and its resistance to conventional antibiotics [3]. Despite the bacillus could stay dormant in the human body without any symptoms for a long time, injury of immune function may induce its reactivation [10].

Urogenital TB is the second common form of extrapulmonary TB which occurs in 15% to 20% cases of pulmonary TB with a prevalence of 400 per 100,000 population, mostly affecting middle-aged men [2, 12]. Given the high prevalence of TB worldwide, urogenital TB reflects a large burden of urogenital diseases, especially in countries with a severe epidemic situation including China. Male genital TB is a rare subtype of urogenital TB, usually occurring in men aged 30–50 years [5]. Similarly, our cohort was consisted of a wide range of ages with a mean age of 50.77 ± 16.1 years (range, 13–90 years). In our study, 10 patients (12.3%) reported a history of TB compared with 34% to 76% reported in the literature [5]. The low rate in our series was probably due to the retrospective nature of our study.

Some studies suggest that male genital TB often results from direct infection from urine, while other experts suggest that haematogenous and lymphatic spread are the most common pathways of initial infection in male genital TB [2, 5]. Muneer et al.[13] thought that TBEO is caused by direct spread from the lower urinary tract or retrograde spread of *M. tuberculosis* via the prostate and into the epididymis, and TB of the testis is always secondary to infection of the epididymis. However, in our study, 23 patients (28.4%) were defined as isolated testicular TB and no patients were diagnosed with prostatic TB, which probably attributed to the latent presentation and delayed diagnosis of TB. In addition, sexual transmission is thought to be possible since *M. tuberculosis* has been isolated form the ejaculate of men with prostatic TB [5, 13].

The onset of clinically evident TBEO is insidious, with variable clinical manifestations. Most studies found that a scrotal swelling, scrotal pain and irritative voiding symptoms are the common initial symptoms of patients with TBEO, which is similar to our findings [4, 8, 12]. On physical examination, the scrotal mass may be either painful or painless [6]. Nonspecific constitutional symptoms of TB such as fever, weight loss, fatigue and night sweats are uncommon [9, 13]. However, suspicion of concomitant TB outside the urogenital tract should arise when these constitutional symptoms are present, such as pulmonary TB [13]. Compared with other reports in the literature, the average duration between onset of the symptoms and the definite diagnosis (mean 6.42 ± 7.0 months, range 0.3–36 months) in our study was longer, probably due to the insidious and asymptomatic onset of TBEO [5, 14–16].

Urinalysis was reported abnormal in 77–90% of patients with urogenital TB [5]. Altiparmak et al.[9] reported that hematuria and pyuria were detected in 79.7% and 67.1% of patients, respectively. However, in our study, pyuria and hematuria were only observed in twenty-two (27.2%) and eight patients (9.9%), respectively. Similar to our findings, abnormal urinalysis (hematuria and pyuria) was detected in 59.6% of patients in another cohort study with 47 cases of epididymal tuberculosis [4]. This result may be because all patients in our study were isolated TBEO without renal involvement. Urine AFB culture was long considered the gold standard in diagnosis of urogenital TB. However, low sensitivity of culture was reported in the literature and the negative urine cultures do not rule out the possibility of TBEO. In addition, cultures may take several weeks to show a delayed result [5, 6, 17]. In our series, however, results of urine AFB cultures were only available in 16 patients since the retrospective nature of the study, and all were negative. On the other hand, patients might not receive the urine AFB cultures when the clinical diagnosis of tumor was considered before surgery. Given the small sample size, our results are not enough to suggest the low significance of urine AFB culture in diagnosis of TBEO.

Recent years, as a rapid test for detecting *M. tuberculosis* DNA and rifampicin resistance, GenXpert MTB/RIF was identified to be the better choice in diagnosing urogenital TB according to its higher sensitivity compared with urine microscopy and culture [13]. Furthermore, interferon-γ release assays (including QuantiFERON-TB Gold In-Tube and T-SPOT TB) were recommended to detect latent TB infection, especially for asymptomatic individuals with high risk of TB infection [13]. However, data of GenXpert MTB/RIF and interferon-γ release assays were not available in our study due to the retrospective design. In addition, some studies reported that nucleic acid amplification (NAA) tests of the urine are helpful adjunctive tools for rapid diagnosis of renal TB, with a specificity and sensitivity of 95.6% and 98.1%, respectively. But the value of NAA tests in diagnosis of male genital TB is still controversial [5, 6]. According to some reviews, FNAC can be used to diagnose TB of the external male genitals [2, 13]. In our opinion, however, FNAC should not be used for diagnosis of TBEO or testicular tumor, given the risk of fistula formation and spread of *M. tuberculosis* or tumor cell. Therefore, none of our patients received FNAC.

**Fig. 2:**
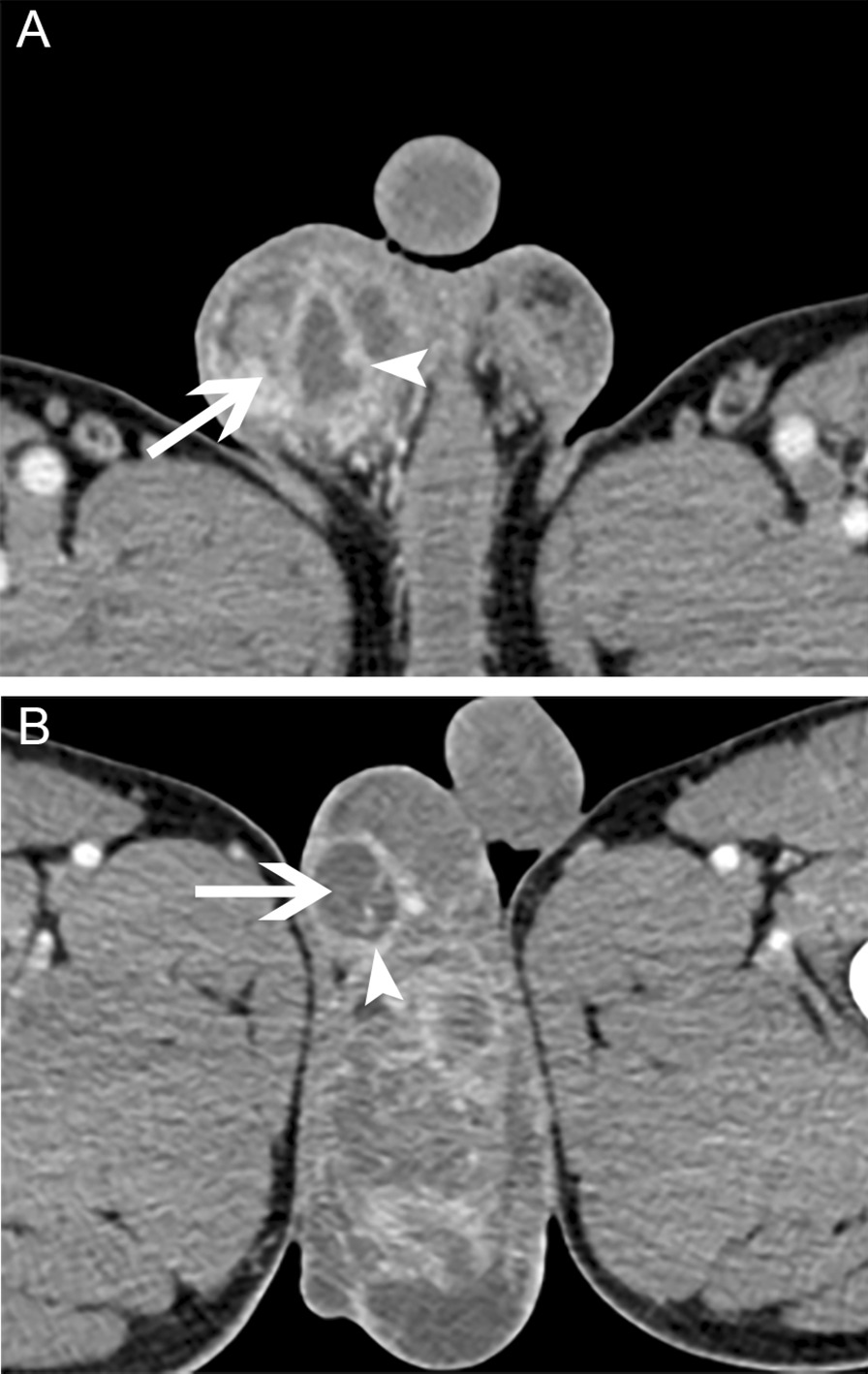
Scrotal Contrast-enhanced CT Scan Images. A contrast-enhanced CT scan showed asymmetric enlargement of the scrotum, in which the irregular mass or nodules (**A**, arrow), cystic lesions (**B**, arrow) and heterogeneous or annular enhancement (arrowheads) were observed

TBEO in the early stages always has no specific scrotal imaging findings [5, 13]. Ultrasound of TBEO can show diffusely or nodular enlarged hypoechoic lesions. Other features including scrotal wall and tunica albuginea thickening, hydrocele, varicocele and intratesticular abscesses can also be seen in scrotal ultrasound. On a contrast-enhanced CT scan, TBEO can be seen as heterogeneous or annular enhancement, cavitation lesions or irregular mass (Fig. 2). Calcification may also be observed in advanced TB. However, these findings in scrotal imaging are not TB-specific and cannot be distinguished from an abscess or malignancy [2, 5, 13]. In addition, the proportion of cases with active pulmonary TB in our study (7.4%) was consistent with the literature [9, 13].

Male TBEO can present as a nonspecific epididymo-orchitis and testicular mass in clinical features that is difficult to differentiate from nonspecific infectious diseases and malignancy [5]. Similarly, scrotal tumors (34.6%) and nonspecific bacterial epididymo-orchitis (28.4%) were the most common misdiagnosis in our study. Given the deficiency of preoperative diagnostic method with high sensitivity and specificity, histopathology of surgical specimens including microscopy, acid fast stain and/or PCR for *M. tuberculosis* remains the gold standard for diagnosis of TBEO, especially in isolated TBEO without renal and prostate involvement [2, 8]. Borges et al. reported a case of TBEO recently, in which a surgical intervention of right epididymo-orchidectomy through the inguinal canal was performed, given the possibility of malignant neoplasm of the epididymis [18]. As reported by a review of urogenital TB, in up to one-fifth of patients, TBEO is only diagnosed after epididymo-orchidectomy and histopathological examination [13]. In our study, all definitive diagnosis of TBEO was confirmed by histopathology of surgical specimens.

Most studies recommended anti-tuberculosis chemotherapy as the first-line treatment for TBEO. Surgery should only be considered for patients not respond to chemotherapy and for the correction of complications [2, 5, 8]. However, the value of anti-tuberculosis chemotherapy alone for TBEO is limited in our study since most patients have developed with complications (hydrocele, abscess, sinus or fistula) or been advanced stages when diagnosis of TBEO was confirmed due to its latent presentation. Moreover, successful medical treatment of TB might be hampered by drug tolerance of *M. tuberculosis *[19]. Recently, Goossens et al. have proposed four possible mechanisms for drug tolerance of *M. tuberculosis*, including metabolic slowdown through reducing the metabolism and growth rate, metabolic shifting, cell wall thickening, and the upregulation of efflux pumps [19]. In our series, 15 patients (18.5%) had received regular anti-tuberculosis pharmacotherapy for 1–12 months in other medical centers before surgery but the scrotal tuberculosis lesions were not controlled effectively, and tuberculous complications including hydrocele, abscess and sinus were observed in 13 patients (16.0%). In this situation, radical surgery is often unavoidable, which can be used for resection of the lesions and histopathological examination [4, 6, 13, 20]. However, it is worth noting that Bedi et al. has successfully treated a 38-year-old patient with TBEO by standard anti-tuberculous medications [14]. Besides, Abraham et al. also reported an Ecuadorian man with TBEO who was cured with 6 months of drug therapy and no surgery was required [21]. Therefore, for patients at early stages without server complications, standard anti-tuberculosis chemotherapy is still necessary to avoid surgical resection [15, 16, 22, 23].

In our study, all patients clinically diagnosed with TBEO received triple therapy of preoperative anti-tuberculosis chemotherapy for 2–4 weeks, radical surgery and postoperative anti-tuberculosis therapy with 4 drugs (rifampicin, isoniazid, ethambutol and pyrazinamide) for 6–12 months. The preoperative anti-tuberculosis chemotherapy was aimed to control the quantity of *M. tuberculosis* in tissues and blood to prepare for surgery, while the postoperative anti-tuberculosis drug therapy was used for eradicating the remaining *M. tuberculosis* to prevent recurrence. Given TB of the testis is always secondary to infection of the epididymis, epididymectomy (33.3%) and epididymo-orchidectomy (64.2%) were the main surgical procedure for TBEO in our study. But there were still two patients (2.5%) who received simple orchiectomy due to the preoperative imaging suggested that the lesion was confined in testis and no epididymal involvement was found during operation. After standard therapy was completed, all patients recovered well and no recurrence was observed during follow up except one patient died from liver cirrhosis. Postoperative complications including sexual dysfunction and sterility were respectively reported in nine (11.1%) and seven (8.6%) patients, suggesting that physicians should pay more attention to the sexual function and fertility of TBEO patients who received radical surgery during follow up.

We must acknowledge several limitations of our study. Firstly, patient data such as serum ESR, CRP and gonadal hormone levels, urine AFB cultures, PCR for *M. tuberculosis*, GenXpert MTB/RIF and interferon-γ release assays were not completed because of the nature of retrospective study. Secondly, the data of patients successfully treated with anti-tuberculosis drugs without surgery were not available for comparative analysis. Thirdly, our study was a single-center study without sufficient data from other medical centers, which may have resulted in a certain degree of selection bias. Finally, some patients lost to follow-up even if try to contact with their relatives and families, which may affect the accuracy of our findings.

## Conclusions

Given the deficiency of diagnostic tools with high sensitivity and specificity, radical surgery followed by histopathological examination might be unavoidable for diagnosis and treatment. We recommend patients diagnosed with advanced TBEO to receive triple therapy of chemotherapy-surgery-pharmacotherapy to correct complications and minimize the risk of recurrence especially in endemic area. After triple therapy is completed, physicians should pay more attention to patients’ sexual function and fertility during follow up.

## Data Availability

The datasets used and/or analyzed in the current study are available from the corresponding author upon reasonable request.

## Abbreviations

TBEO: Tuberculous epididymo-orchitis
TB: Tuberculosis
ESR: Erythrocyte sedimentation rate
CRP: C-reactive protein
PCR: Polymerase chain reaction
AFB: Acid fast bacilli
FNAC: Fine-needle aspiration cytology
β-HCG: Beta-human chorionic gonadotropin
AFP: Alpha-fetoprotein
LDH: Lactate dehydrogenase
SD: Standard deviation
IQR: Interquartile range
CT: Computed tomography
MRI: Magnetic resonance imaging
MTBC: Mycobacterium tuberculosis complex
NAA: Nucleic acid amplification
